# Contraception pathway: application for midlife women

**DOI:** 10.1186/s40695-017-0029-3

**Published:** 2017-10-24

**Authors:** Chi-Son Kim, Deanna Tikhonov, Lena Merjanian, Adrian C. Balica

**Affiliations:** Department of Obstetrics, Gynecology and Reproductive Sciences, Rutgers Robert Wood Johnson Medical School, 125 Paterson Street, New Brunswick, NJ 08901 USA

**Keywords:** Contraception in midlife women, Unintended pregnancy, Contraceptive pathway

## Abstract

**Objective:**

To create a system where evidence based medicine can be applied to accommodate every woman’s needs by designing a contraceptive pathway that can be utilized by any healthcare provider, regardless of the patient’s age, and to offer appropriate counseling in order to maximize patient outcomes, especially for the midlife woman.

**Methods:**

United States Medical Eligibility Criteria for Contraceptive Use, 2016 (US MEC) was used as the framework for these recommendations for a contraceptive care pathway that can be incorporated into care for midlife women.

**Discussion:**

By utilizing a total office approach that includes the scheduler, receptionist, medical assistant, nurse and health care provider as members of a team, the entire spectrum of the patient population in need of contraception from teenagers to midlife can be captured. Specifically for midlife women the need for an effective form of contraception may be overlooked as fecundity declines in this age group. This paper will highlight the use of this pathway for midlife women.

## Background

In 2011, 45% of the 6.1 million pregnancies were unintended in the United States (U.S.) [1]. Fortunately the overall unintended pregnancy rate has declined over the recent years from 54% in 2008 to 45% in 2011 [1]. Nevertheless, about half are still unintended, emphasizing the importance of appropriate contraceptive counseling. Specifically, the group that is often overlooked when discussing contraception is midlife women. Many of the choices in family planning for this group of patients may not be ideal for them due to comorbidities that put them at an increased risk for poor health outcomes, such as cardiovascular disease or chronic hypertension.

Despite a number of new and innovative options approved by the FDA over the last fifteen years, many have not directly addressed the needs of the midlife woman. Healthcare providers are struggling not only with recommending the most effective form of contraception, but also with overcoming social biases and myths about midlife women. The most significant bias concerns a woman’s diminished fertility which can cause the provider to overlook the potential risk for an unplanned pregnancy [2]. It is important that all women of reproductive age, through one year past their last normal menstrual period be given contraceptive counseling if they are at risk for unintended pregnancy.

To best address the needs of the midlife woman, as well as all reproductive- aged women, we created a contraceptive pathway that can be utilized by any healthcare provider to identify a woman’s specific needs, regardless of her age, and to offer appropriate counseling in order to maximize patient outcomes. Obstetrician/gynecologists, family practice providers, pediatricians, advanced practice nurses, nurse midwives and primary care providers can apply this pathway to meet the specific needs of their patient population. This clinical pathway, is a structured care plan to detail essential steps in the care of the patient with a specific clinical problem, such as the midlife woman [3]. Both clinical staff and providers are involved in the pathway to ensure a smooth process for the patient to obtain the most effective and appropriate method. Based on a patient’s age and medical history this pathway can be tailored to meet their contraceptive needs. Standardized delivery of clinical care has become central in evidence based medical practice.. The development of a clinical pathway is an example of a strategy created to reduce variation in clinical care, but still take into account patient differences [2].

A key component of the clinical pathway presented in this article is the ability to utilize the entire office staff creating a clinical care team in order to individualize care and deliver it seamlessly [4]. The team includes the schedulers, receptionists, medical assistants, LPNs and RNs, and healthcare providers. Patient care teams have become a cornerstone in patient centered care illustrated within the Veterans Affairs clinics using Patient Aligned Care Teams or PACT [5]. Effective contraceptive counseling requires not only access to the latest evidence based medicine, but also having a team understand each woman’s needs and circumstances, providing seamless coordination [4]. For example, in a scenario involving a midlife patient who smokes 1 pack of cigarettes per day and who has migraine headaches, her options may be limited. A contraceptive pathway can be applied to accommodate this woman’s needs, so that the best contraceptive choice is made. In addition, health care providers from various specialties can utilize the same pathway improving contraceptive access for the patient regardless of where she receives her care, therefore enhancing patient safety.

## Methods

We will describe our clinical pathway and how to utilize it when caring for midlife women. The contraceptive clinical pathway we suggest encompasses the United States Medical Eligibility Criteria for Contraceptive Use, 2016 (US MEC) guidelines [6]. The US MEC was created by the CDC to guide healthcare providers in counseling women, men, and couples about contraceptive choices [6]. Although midlife women are included, those women without a menses for one year, indicating menopause, are excluded [7].

### Overview

**Fig. 1:**
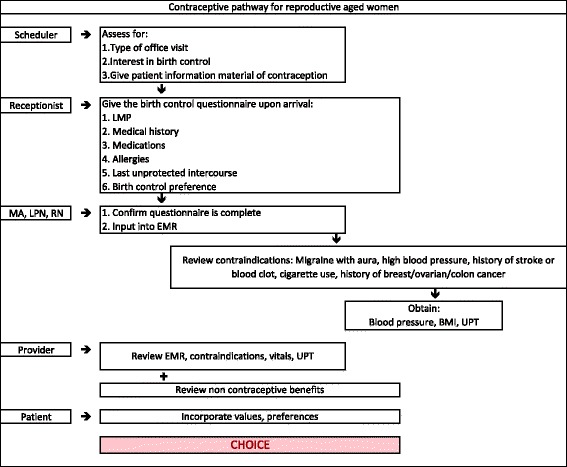
Contraceptive pathway for reproductive aged women

**Fig. 2:**
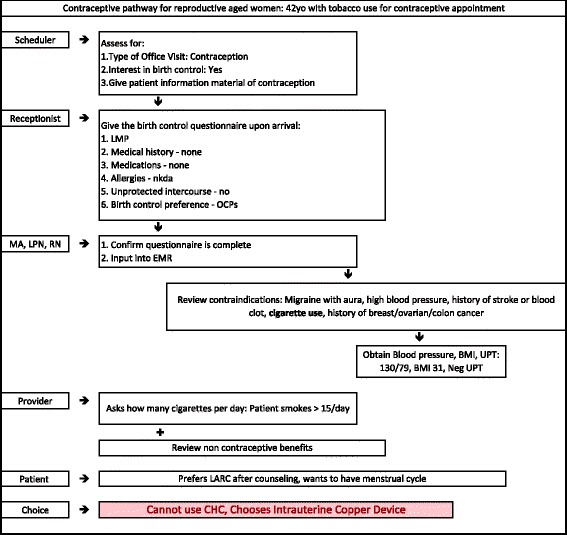
Contraceptive pathway for reproductive aged women: Example of a patient who is a smoker over age 35

**Fig. 3:**
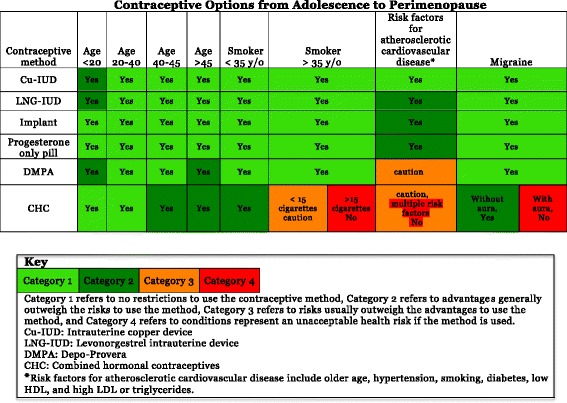
Contraception graph based on age and common comorbidities found in midlife women based on US MEC 2016^5^

To use this pathway, every patient encounter should be viewed as an opportunity to discuss contraceptive options. The clinical care team will be involved throughout the process to coordinate women’s contraceptive care. As seen in Fig. 1, the contraceptive care pathway starts with the patient scheduling an appointment with a scheduler who will assess if she is interested in a birth control method and provide her with a resource for contraceptive information to review prior to her appointment. This component of the care pathway creates a great opportunity for the patient to learn about her contraceptive options and potentially narrow her choices prior to the visit. When the patient arrives to the office, the receptionist will provide a birth control questionnaire for her to complete. This action by the receptionist enhances the flow of the visit because the questionnaire begins to tailor the contraceptive options for which the patient is eligible. The medical/nursing staff will confirm the questionnaire is complete, input the information into the electronic medical record, and obtain vital signs from the patient. They will also utilize a checklist, which assesses for contraindications, as outlined in Figs. 1 and 2. After these activities are completed, the health care provider will review the patient’s electronic medical record and meet with the patient. The pathway ends with the counseling and contraceptive choice determined in conjunction by the patient and healthcare provider. Figure 2 illustrates the application of the contraceptive care pathway to a woman who is 42 years old and has a history of tobacco use. Clinicians using this care pathway can take into account varying needs and circumstances of each age group, referring to the U. S. Medical Eligibility Criteria for Contraceptive Use shown in Fig. 3.

For optimal care, phone or patient portal follow up is performed at three months and one year by a nurse to identify the following outcomes: contraceptive failures that lead to pregnancy, continued use of contraceptive method, patient satisfaction with the method, and adverse events that may have caused discontinuation of method. The outcomes will be recorded by the nurse and compared to patients within the practice who are not using this contraceptive pathway to note the difference in outcomes. The clinical care team will meet for huddles throughout the follow up process to review the outcomes being revealed and discuss any challenges they have encountered using the contraceptive clinical pathway. These data will be used as an educational tool for quality improvement to determine if the utilization of this pathway improves unintended pregnancy rates, compliance with birth control method, and improves patient safety.

The contraceptive clinical pathway specifically benefits midlife women because it reminds the clinical care team of the importance of addressing contraceptive needs and reassessing for contraindications in women who may not believe they can become pregnant and have comorbidities. It also allows for better opportunity to discuss contraceptive change. For example, a 42 year old woman who is found to have a blood pressure of 170/100 during her well woman exam, may have been using combined oral contraceptive pills for several years and ask for a refill to continue. The contraceptive pathway ensures that her contraindications will be reviewed and allows for a dialogue between the patient and provider to reach the safest most acceptable method for the patient. Specific points geared to midlife women that practitioners should focus on include [7]:

1. future pregnancy desires, including whether they will pursue ART
2. risk for sexually transmitted infections if she has recently terminated a committed relationship
3. medical conditions including cardiovascular disease, stroke, VTE, liver disease, gall bladder symptoms
4. gynecologic conditions such as fibroid uterus, heavy menstrual bleeding, endometriosis
5. lifestyle factors such as smoking, obesity, and frequent air travel

## Discussion

The challenge in contraceptive management for midlife women differs from that for other reproductive aged women. Pregnancy rates have increased for women aged 30 and older since the late 1990s, especially for those aged 40 and over. This trend is mainly due to delayed first and second births, but there are also unplanned and unintended pregnancies [1]. The data suggest that the unintended pregnancy rate for women 35 and older is 16%, accompanied by the incidence of abortion increasing from 1998 to 2008 [1, 8]. Regarding contraception, U.S. women in this age group have a higher prevalence of sterilization when compared to Canada and the United Kingdom (UK) [8]. The need for an effective form of contraception may be overlooked in midlife women since fecundity is diminished in this age group [7]. In addition, with increase in irregular cycles, ovulation is less predictable, making techniques such as the rhythm method difficult to use effectively. Potential clinical and social consequences of an unintended pregnancy for midlife women are usually more detrimental to their health and their fetus’ health as well, when compared to younger reproductive-aged women. Risks associated with pregnancy in women over 40 include miscarriage, aneuploidy, maternal morbidity and mortality, and neonatal complications including preterm delivery [9].

Although age alone is not a contraindication for any specific contraceptive method, it should again be emphasized that midlife women often have comorbidities that must be considered when deciding on a form of contraception. The USMEC categorizes eligibility for use of different contraception into four categories: Category 1-no restriction (method can be used); Category 2-advantages generally outweigh theoretical or proven risks; Category 3-theoretical or proven risks usually outweigh the advantages; and Category 4- unacceptable health risk (method not to be used) [6]. The common comorbidities that midlife women should be routinely screened for include: hypertension, obesity, uncontrolled diabetes, ischemic heart disease, and multiple risk factors for cardiovascular disease (smoking, obesity, diabetes, hyperlipidemia, and hypertension) [6]. For this age group, despite these increased risk factors, the benefit of using most contraceptives outweigh the risk of unintended pregnancy.

Starting with the most effective birth control method, long acting reversible contraceptives (LARCs) include the copper IUD, the levonorgestrel-releasing IUD, and the subdermal etonogestrel implant. There are different non-contraceptive health benefits for these methods as well. The copper IUD also can be used as an emergency contraceptive, in addition to reducing the risk of endometrial cancer. This method is hormone free, making it an ideal option for midlife women with multiple comorbidities [10]. The levonorgestrel-releasing IUD’s health benefit includes reduction in heavy menstrual bleeding, including bleeding associated with leiomyoma and adenomyosis, hence improving quality of life [9, 11] In the UK, the levonorgestrel-releasing IUD also is approved for endometrial hyperplasia protection during estrogen therapy [9]. The subdermal implant causes variable bleeding pattern, but it has been shown to reduce dysmenorrhea [9].

The combined hormonal contraceptives (CHC) are available as daily pills, a monthly vaginal ring, and weekly transdermal patch. Indications and contraindications are similar for all combined hormonal methods for all age groups [12]. Current guidelines suggest use of combined hormonal contraceptives in midlife women without contraindications [6]. CHCs not only provide contraceptive benefits but also offer cycle regularity, treatment of vasomotor symptoms, protection against bone loss, and reduced risk of ovarian and endometrial cancer [11, 13].

Barrier methods including the male and female condom, are forms of contraception that can be used by most people regardless of age or comorbidities. Furthermore, they are the only methods that also provide protection against sexually transmitted infections. Research has noted that sexually transmitted infections are still a prevalent issue among midlife women and are often underestimated among this population [7].

## Conclusions

On average, U.S. women have two children. This means that a woman will spend about three years of her life trying to conceive, being pregnant or postpartum. The rest of a woman’s life is then spent on avoiding unintended pregnancy [1]. Of women aged 15–44 who have ever had sexual intercourse, 99% of them have used at least one contraceptive method [10]. Appropriate contraceptive counseling, leading to the use of most effective method of contraception and adherence is crucial, and should always include the midlife woman. Despite misconceptions that certain contraceptive methods cannot be used in certain age groups, we highlight that age alone is not a contraindication to most birth control methods [6].

Additionally, midlife can be seen as a difficult time for contraceptive management among providers as patients may have comorbidities or patients may not view contraception as an important need with their declining fecundity. However, unintended pregnancy among older women has far worse implications for a woman’s health than utilizing a contraceptive method in most circumstances. It also is important to note most contraceptive methods have non-contraceptive health benefits that could be of value to the midlife patient.

Contraceptive counseling should include specific considerations for midlife women. Our clinical contraceptive care pathway is a tool to be used in conjunction with USMEC to guide healthcare providers. The goal of the contraceptive pathway is to incorporate a total office approach using a clinical care team that is patient- driven. Individual preferences and medical history are taken into account to help navigate evidence-based medicine in helping midlife women, as well as all other reproductive-aged women, choose the most effective and appropriate form of contraception. This pathway strives to let clinicians integrate the USMEC in contraceptive care.
